# Accumulation, morpho-physiological and oxidative stress induction by single and binary treatments of fluoride and low molecular weight phthalates in *Spirodela polyrhiza* L. Schleiden

**DOI:** 10.1038/s41598-019-56110-w

**Published:** 2019-12-27

**Authors:** Ritika Sharma, Arpna Kumari, Sneh Rajput, Nishu, Saroj Arora, Rajkumar Rampal, Rajinder Kaur

**Affiliations:** 1 Department of Botanical and Environmental Sciences, Guru Nanak Dev University, Amritsar, 143005 Punjab India; 2 Department of Environmental Science, University of Jammu, Jammu, 180016 India

**Keywords:** Plant sciences, Ecology

## Abstract

The present study examined the interactive effects of fluoride and phthalates on their uptake, generation of reactive oxygen species and activation of antioxidative defence responses in *Spirodela polyrhiza* L. Schleiden. A hydroponic study was conducted in which *S. polyrhiza* cultured in Hoagland’s nutrient medium, was exposed to fluoride (50 ppm) and different concentrations viz., 75, 150 300 ppm of diethyl phthalate (DEP) and diallyl phthalate (DAP) individually as well as in combination for the time period of 24, 72, 120 and 168 h respectively. A significant decline in fresh weight, dry to fresh weight ratio, total chlorophyll, carotenoid content and increased anthocyanin content was observed. Fluoride and phthalates was found to be readily accumulated by *S. polyrhiza* in all the exposure periods. Interestingly, when binary treatments were given in nutrient medium, uptake of both fluoride and phthalate was found to be influenced by each other. In combined treatments, DEP stimulated fluoride uptake, while its own uptake was restricted by fluoride. In contrary to this, fluoride stimulated DAP uptake. Moreover, combined stress further caused significant decrement in carbohydrate, protein content and increment in MDA levels, phenolic content and electrolyte leakage. Nevertheless, phthalates showed more pronounced oxidative stress and growth inhibition compared to fluoride. To cope up with the oxidative damage, enhanced level of antioxidant enzymatic activities was observed in *S. polyrhiza* under both fluoride and phthalate stress as compared to control. Scanning electron microscope imaging of leaf stomata revealed that combined stress of fluoride with phthalates caused distortion in the shape of guard cells. Confocal micrographs confirmed the generation of reactive oxygen species, cell damage, disruption in membrane integrity, and enhanced levels of glutathione in plant cells. This study focussed on ecotoxicological and interactive significance of fluoride led phthalate uptake or vice versa which was also assumed to confer tolerance attributes.

## Introduction

In today’s modern world, the widespread use of inorganic and organic compounds for agricultural and industrial purposes has induced dramatic influence on physical, chemical and biological characteristics of environment. Moreover, alterations in growth, physiology, plant species abundance and productivity is also a consequence of toxic effects of such contaminants^1^. Detrimental effects on living organisms gives us an alarming indication, and provides a powerful approach for interpreting the effects of contaminants, subsequently preventing environment contamination and human diseases^2^. Though, assessment of cumulative effects of environmental pollutants on living organisms is often difficult, as limited information is available on the combined effects of some stressors in the literature. Besides, maximum risk assessments of polluted environment by these contaminants are based on the guideline values derived from their ecotoxicological properties. Fascinatingly, interaction of environmental stressors induces additive, synergistic and sometimes antagonistic effects on living organisms. Therefore, it is necessary to unravel the ecotoxicological and combined effects of stressors on specific living organisms which act as an early warning bioindicators^1,3–5^.

In this present investigation, we analysed the individual and joint toxic effects of fluoride and two phthalates, namely DEP and DAP on physiological and biochemical attributes of *S. polyrhiza*. Both fluoride and phthalates are known for their deleterious effects on environment and human health. Over the last few decades their improper use in industrial as well as agricultural sector contributed to environmental pollution. Among them, fluoride (F) is a common environmental pollutant released to the environment by various industrial sources, agricultural activities, and weathering of volcanic ashes^6–8^. It has led to the pollution of many areas and exacerbated human health, whereas phthalate esters (PEs) are known as plasticizers that are widely used as additives in plastic products with the overall aim of enhancing flexibility and durability^9^. Since, PEs are unable to bind covalently to the plastic which force them to leach out into the environment and thus, deteriorate the environmental health. Moreover, their tendency to bioaccumulate in living organisms induced endocrine disruption, malformations and reproductive disorders which is a matter of serious concern^10^. Both these environmental stressors tend to accumulate in soil, air and water and induce adverse effects. Consequently, living organisms are affected by both fluoride and phthalates *via* food chain. Contamination of water with fluoride and phthalates has been detected worldwide due to anthropogenic, industrial and agricultural activities. Living organisms are affected *via* consumption of contaminated water or food. Aquatic organisms are influenced directly^11^. Overall, they pose serious threats to terrestrial as well as aquatic plants and animals.

Plants may encounter with different abiotic stresses during their lifespan and show varied responses to the stress. They interfere with the normal cellular and metabolic functions of plant cells and disturb homeostasis along with the accumulation of reactive oxygen species (ROS)^12^. To counterbalance, plant stimulate the activation of various antioxidant enzymes including superoxide dismutase (SOD), catalase (CAT), peroxidases like ascorbate peroxidase (APX) and guaiacol peroxidase (GPOX) as well as glutathione reductase (GR). Along with this, plants have potential to accumulate, sequester, biotransform or remove the contaminants. Use of aquatic plants for the removal of pollutants from polluted water has gained so much importance nowadays. Among them, duckweed species served as a good option. Previous researches also reported the efficient role of duckweed species in the removal of heavy metals^13^, pesticides^14^ and other xenobiotics from the contaminated water. Therefore, *Spirodela polyrhiza* has been chosen as an effective tool for the biomonitoring of the cumulative effects of fluoride and phthalates.

*Spirodela polyrhiza*, commonly known as duckweed, is a free-floating aquatic macrophyte belonging to a Lemnaceae family that flourishes in quiescent water. Being a major producer, *S. polyrhiza* is the first aquatic organism susceptible to a variety of inorganic and organic pollutants, thus function as an effective bioindicator of contaminants in ecotoxicological research^11,15^. Considering all this, our present investigation envisaged the toxic and interactive effects of fluoride and phthalates, namely DAP and DEP on *Spirodela polyrhiza* L.

To the best of our knowledge, till date no study has been undertaken to investigate the interactive effects of fluoride and phthalates on any plant and this is the first report which is reporting the combined effects of fluoride and phthalates on growth, physiological and biochemical parameters of *S. polyrhiza*. Additionally, this study is a dedicated attempt in this perspective to investigate tolerance and defense strategies of *S. polyrhiza* to combined effect of fluoride and phthalates on growth parameters, accumulation of fluoride and phthalates content, pigments (chl a, chl b, total, carotenoids and anthocyanins), carbohydrate, protein, lipid peroxidation, proline, electrolyte leakage, phenols, antioxidant enzymatic activities (SOD, CAT, APX, GPOX and GR), stomatal movements and cell viability.

## Results

### Analysis of phthalates

Chromatographic separation of mixed sample containing DEP and DAP was shown in **(**Supplementary Fig. 1**)** where two peaks were obtained separated from baseline and without any interference peak. Total run time was 20.01 min and the retention time (RT) of DEP and DAP was 4.52 and 5.44 min, respectively.

### Method validation

As shown in Table 1(b) good linearities were obtained over the range of 5–400 mg L^−1^ with correlation coefficients (R^2^) of 0.99 for both the phthalates. Mean recovery percentage for DEP and DAP was 99.15% and 98.15% with RSD 0.28 and 0.18 respectively. The LODs for DEP and DAP were found to be 1.90 and 0.75 µg L^−1^ and LOQs for each DEP and DAP were 5.77 and 2.30 µg L^−1^, respectively **(**Supplementary File S1**)**.

**Table 1:** Accumulation of DEP, DAP and fluoride content by *S. polyrhiza* at different exposure periods.ou.

Treatment	Duration	DEP content (mg/kg fw)	Fluoride content (mg/kg dw)	DAP content (mg/kg fw)	Fluoride content (mg/kg dw)
Control 1	24 hours	ND	ND	ND	ND
Control 2		ND	ND	ND	ND
50 ppm F^−^		ND	195.2 ± 0.81a	ND	192.4 ± 2.4a
75 ppm DEP/DAP		76.73 ± 0.3e	ND	364.13 ± 2.46c	ND
150 ppm DEP/DAP		77.28 ± 0.42e	ND	325.18 ± 4.88d	ND
300 ppm DEP/DAP		142.02 ± 1.68a	ND	201.06 ± 11.01e	ND
50 ppm F^−^ + 75 ppm DEP/DAP		132.52 ± 0.5b	270 ± 40.84ab	312.94 ± 1.17d	138 ± 6.92a
50 ppm F^−^ + 150 ppm DEP/DAP		111.05 ± 0.37d	180.8 ± 14.66ab	898.1 ± 2.74a	26.6 ± 3.29b
50 ppm F^−^ + 300 ppm DEP/DAP		118.98 ± 0.12c	121.6 ± 16.44b	822.94 ± 2.35b	17.6 ± 0.87b
Control 1	72 hours	ND	ND	ND	ND
Control 2		ND	ND	ND	ND
50 ppm F^−^		ND	571 ± 13.9a	ND	551 ± 0.2a
75 ppm DEP/DAP		78.74 ± 0.74c	ND	659.73 ± 23.73a	ND
150 ppm DEP/DAP		80.32 ± 2.09c	ND	616.6 ± 2.11a	ND
300 ppm DEP/DAP		80.6 ± 3.07c	ND	194.53 ± 2.61c	ND
50 ppm F^−^ + 75 ppm DEP/DAP		95.26 ± 10a	519.8 ± 14.02a	276.37 ± 7.2b	156.6 ± 17.32b
50 ppm F^−^ + 150 ppm DEP/DAP		87.26 ± 14.53b	182.1 ± 2.59b	60.82 ± 0.04d	71.6 ± 1.21c
50 ppm F^−^ + 300 ppm DEP/DAP		79.12 ± 0.5c	170 ± 4.25b	52.75 ± 0.37d	78 ± 0.69c
Control 1	120 hours	ND	ND	ND	ND
Control 2		ND	ND	ND	ND
50 ppm F^−^		ND	674.4 ± 35.85a	ND	574.4 ± 19.73a
75 ppm DEP/DAP		75.97 ± 0.21c	ND	79.1 ± 0.17b	ND
150 ppm DEP/DAP		89.06 ± 0.05b	ND	64.89 ± 0.23 cd	ND
300 ppm DEP/DAP		99.63 ± 0.13a	ND	64.43 ± 0.26d	ND
50 ppm F^−^ + 75 ppm DEP/DAP		65.22 ± 0.63e	369.2 ± 35.85b	88.18 ± 0.28a	48.8 ± 3.29b
50 ppm F^−^ + 150 ppm DEP/DAP		70.66 ± 0.25d	177.6 ± 29.09c	65.96 ± 0.45c	60.6 ± 3.11b
50 ppm F^−^ + 300 ppm DEP/DAP		63.68 ± 0.18 f	55.4 ± 10.56c	49.24 ± 0.23e	61.6 ± 0.87b
Control 1	168 hours	ND	ND	ND	ND
Control 2		ND	ND	ND	ND
50 ppm F^−^		ND	648.4 ± 17.9a	ND	608.4 ± 2.16a
75 ppm DEP/DAP		0.15 ± 0.07a	ND	54.06 ± 0.19a	ND
150 ppm DEP/DAP		2.81 ± 1.32a	ND	50.68 ± 0.16c	ND
300 ppm DEP/DAP		0.14 ± 0.06a	ND	52.43 ± 0.23b	ND
50 ppm F^−^ + 75 ppm DEP/DAP		1.16 ± 0.54a	130 ± 24.33b	52.48 ± 0.24b	33 ± 4.08b
50 ppm F^−^ + 150 ppm DEP/DAP		0.16 ± 0.07a	101.6 ± 4.46b	49.13 ± 0.11e	25.2 ± 0.91bc
50 ppm F^−^ + 300 ppm DEP/DAP		0.22 ± 0.1a	73 ± 1.44b	49.65 ± 0.012de	16.6 ± 0.91c
F-ratio	Two-way ANOVA				
F_F-_ F_DEP/DAP_ F_F-×DEP/DAP_		4383*	42.33*	9683.4*	207.2*
		328*	378.20*	684.04*	3905.03*
		670*	35.12*	1331.068*	215.08*
		3.80	112.35	32.54	33.94
HSD					

Data is shown as (mean ± SE, two-way ANOVA, Tukey’s HSD), * indicates significant at (*p* ≤ 0.05). ND = not detected. Mean values with same letters signifies no significant difference between two values of different concentrations for the same exposure period (using one-way ANOVA).

### Accumulation of phthalate and fluoride content in *S. polyrhiza*

In all the investigation, no accumulation of fluoride, DEP and DAP was found in control samples. The lowest DEP content measured in fronds was obtained after treatment with 75 ppm DEP alone during all the durations, while the maximum content in fronds was observed at the combined treatment of fluoride with 75 ppm DEP after the exposure period of 24 hours. However, minimal accumulation of DEP content by the fronds was recorded after the longer exposure period of 168 hours. On the other hand, individual fluoride content as well as fluoride content in combination with DEP showed significantly enhanced accumulation after all the duration periods **(**Table 1**)**.

Contrary to this, accumulation of DAP content was found more by the plant as compared to DEP, with maximum content was found at the single (150 ppm) and binary combinations (50 ppm F^−^ + 150 ppm) after 24 hours. However, plant accumulated low DAP content at other durations when given combined treatment of 50 ppm fluoride with all other DAP concentrations, which was maximum at the initial combined concentrations (50 ppm F- + 75 ppm) and then further reduced drastically at other proceeding combined concentrations. Notably, accumulation of fluoride content by plant is minimum at all the binary combinations with DAP after all the exposure periods **(**Table 1**)**.

### Growth inhibition

**Figure 1:**
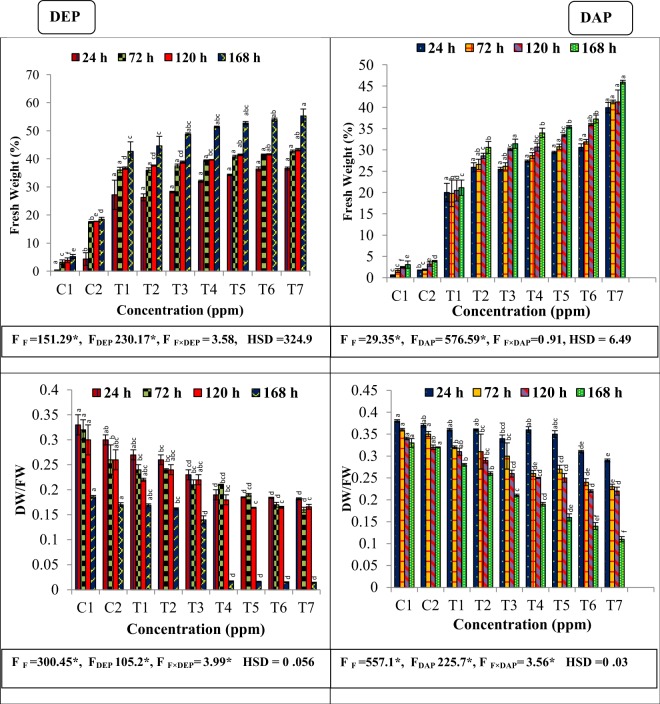
Interactive effects of fluoride and phthalates (DEP and DAP) on fresh weight (%age) and dry to fresh weight ratio (DW/FW) of *S. polyrhiza* exposed for 24, 72, 120 and 168 hours. Treatments: C1 – control, C2-control, T1 – 50 ppm F^−^, T2 –75 ppm DEP/DAP, T3- 150 ppm DEP/DAP, T4 – 300 ppm DEP/DAP, T5 – 50 ppm F^−^ + 75 ppm DEP/DAP, T6 – 50 ppm F^−^ + 150 ppm DEP/DAP, T7 – 50 ppm F^−^ + 300 ppm DEP/DAP. Data is shown as (mean ± SE, two-way ANOVA, Tukey’s HSD), * indicates significant at (*p* ≤ 0.05). Mean values with same letters signifies no significant difference between two values of different concentrations for the same exposure period (using one-way ANOVA).

Significant decline in fresh weight was observed in concentration-duration dependent manner after the application of all the individual as well as combined concentrations of fluoride, DEP and DAP. Combined concentrations led to more decline in fresh weight, with maximum percentage decrease in fresh weight was found to be 55.26% and 45.93% at the highest combined concentration of fluoride with DEP and DAP respectively after 168 h. Similarly, all the applied treatments significantly affected dry to fresh weight ratio of the treated plant with respect to untreated plant at all the durations **(**Fig. 1).

### Effect on photosynthetic pigments

**Figure 2:**
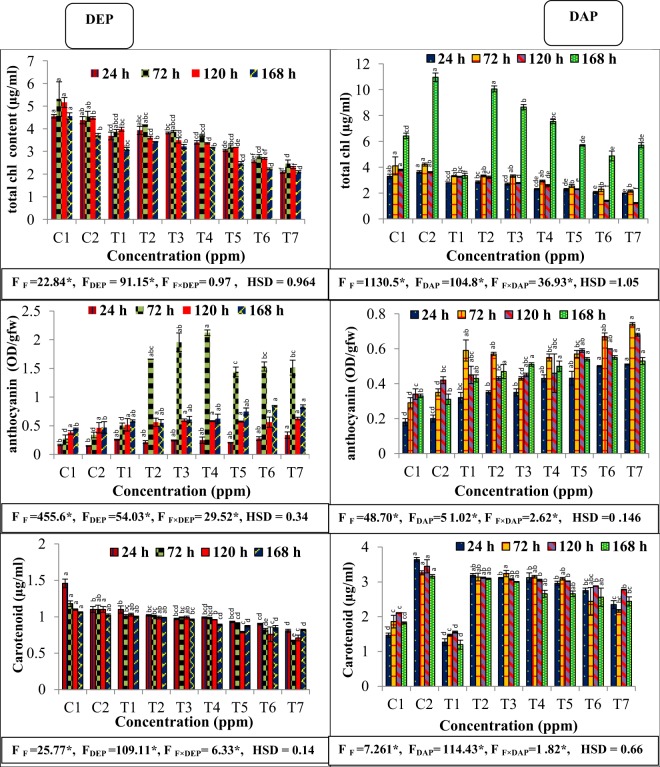
Interactive effects of fluoride and phthalates (DEP and DAP) on total chlorophyll, anthocyanin and carotenoid contents of *S. polyrhiza* exposed for 24, 72, 120 and 168 hours. Treatments: C1 – control, C2-control, T1 – 50 ppm F^−^, T2 –75 ppm DEP/DAP, T3- 150 ppm DEP/DAP, T4 – 300 ppm DEP/DAP, T5 – 50 ppm F^−^ + 75 ppm DEP/DAP, T6 – 50 ppm F^−^ + 150 ppm DEP/DAP, T7 – 50 ppm F^−^ + 300 ppm DEP/DAP. Data is shown as (mean ± SE, two-way ANOVA, Tukey’s HSD), * indicates significant at (p ≤ 0.05). Mean values with same letters signifies no significant difference between two values of different concentrations for the same exposure period (using one-way ANOVA).

In the present investigation, single and binary treatments of DEP and fluoride induced significant decrease in total chlorophyll content as compared to the respective controls during all the durations, but binary treatments of fluoride with 300 ppm DEP showed minimum chlorophyll content. Also, individual as well as combined treatment of fluoride with DAP showed decreasing trend in total chlorophyll content at all the durations and the maximum content was found after 168 h of treatment period (Fig. 2). Negative β-coefficients indicated that treatment of fluoride and phthalate decreased the chlorophyll content to some extent, while negative β- coefficients for interaction of F x phthalate depicted further decrement in the content **(**Table 2**)**.

**Table 2:** Multiple linear regression analysis (MLR) showing interactive effects of fluoride and phthalates on growth and biochemical parameters of Spirodela polyrhiza.

Parameter	Duration (h)	F + DEP					F + DAP				
		MLR equation	β-regression coefficients			Multiple correlation coefficient (r) *(p ≤ 0.05)	MLR equation	β-regression coefficients			Multiple correlation coefficient (r)*(p ≤ 0.05)
			β_F_	β_DEP_	β_F×DEP_			β_F_	β_DAP_	β_F×DAP_	
Fresh weight (%age)	24	*Y* = 12.3 + 0.43*X*_1_ + 0.07*X*_2_−0.001 *X*_1_*.X*_2_	1.02	0.79	−0.70	0.87*	*Y* = 11.04 + 0.27*X*_1_ + 0.06*X*_2_− 0.0004 *X*_1_*.X*_2_	0.62	0.67	−0.19	0.85*
	72	*Y* = 20.06 + 0.28*X*_1_ + 0.04*X*_2_− 0.0005 *X*_1_*.X*_2_	0.99	0.59	−0.38	0.93*	*Y* = 11.25 + 0.29*X*_1_ + 0.007*X*_2_− 0.00005 *X*_1_*.X*_2_	0.65	0.68	−0.22	0.85*
	120	*Y* = 25.52 + 0.30*X*_1_ + 0.06*X*_2_− 0.001 *X*_1_*.X*_2_	0.91	0.78	−0.67	0.81*	*Y* = 13.25 + 0.34*X*_1_ + 0.07*X*_2_− 0.0008 *X*_1_*.X*_2_	0.76	0.70	−0.37	0.84*
	168	*Y* = 28.46 + 0.47*X*_1_ + 0.09*X*_2_− 0.002 *X*_1_*.X*_2_	0.96	0.82	−0.73	0.84*	*Y* = 14.05 + 0.34*X*_1_ + 0.008*X*_2_− 0.0007 *X*_1_*.X*_2_	0.68	0.72	−0.30	0.85*
Dry weight to fresh weight	24	*Y* = 0.29 − 0.02*X*_1_− 0.0004*X*_2_− 0.0000007 *X*_1_*.X*_2_	−1.18	−0.84	0.81	0.96*	*Y* = 0.36 − 0.0001*X*_1_ − 0.00003*X*_2_− 0.000002 *X*_1_*.X*_2_	−0.12	−0.13	−0.68	0.86*
	72	*Y* = 0.25 − 0.001*X*_1_− 0.0002*X*_2_− 0.000006 *X*_1_*.X*_2_	−0.86	−0.50	0.19	0.91*	*Y* = 0.34 − 0.001*X*_1_ − 0.0003*X*_2_ + 0.000003 *X*_1_*.X*_2_	−0.88	−0.76	0.41	0.95*
	120	*Y* = 0.26 − 0.002*X*_1_− 0.003*X*_2_− 0.000006 *X*_1_*.X*_2_	−1.16	−0.68	0.69	0.91*	*Y* = 0.31 − 0.001*X*_1_ − 0.0002*X*_2_ + 0.000002 *X*_1_*.X*_2_	−0.79	−0.68	0.31	0.88*
	168	*Y* = 0.19 − 0.004*X*_1_− 0.0005*X*_2_− 0.00001 *X*_1_*.X*_2_	−1.22	−0.78	0.78	0.97*	*Y* = 0.30 − 0.032*X*_1_ − 0.0004*X*_2_ + 0.000004 *X*_1_*.X*_2_	−0.94	−0.67	0.35	0.97*
Total chlorophyll content (mg/ml)	24	*Y* = 4.3 − 0.02*X*_1_ − 0.003*X*_2_− 0.00002 *X*_1_*.X*_2_	−0.64	−0.42	−0.14	0.97*	*Y* = 3.38 − 0.02*X*_1_ − 0.004*X*_2_− 0.000006 *X*_1_*.X*_2_	−0.98	−0.78	0.55	0.93*
	72	*Y* = 4.4 − 0.02*X*_1_ − 0.003*X*_2_− 0.000008*X*_1_*.X*_2_	−0.73	−0.40	−0.06	0.96*	*Y* = 3.92 − 0.03*X*_1_ − 0.004*X*_2_− 0.00005 *X*_1_*.X*_2_	−0.95	−0.58	0.33	0.93*
	120	*Y* = 4.2 − 0.02*X*_1_ − 0.003*X*_2_− 0.00001 *X*_1_*.X*_2_	−0.58	−0.52	−0.01	0.94*	*Y* = 3.50 − 0.02*X*_1_ − 0.003*X*_2_− 0.000003 *X*_1_*.X*_2_	−0.89	−0.65	0.29	0.96*
	168	*Y* = 3.61 − 0.02*X*_1_ − 0.002*X*_2_− 0.000004 *X*_1_*.X*_2_	−0.88	−0.30	−0.31	0.96*	*Y* = 10.8 − 0.11*X*_1_ − 0.01*X*_2_− 0.0002 *X*_1_*.X*_2_	−1.24	−0.55	0.59	0.97*
Carotenoid content (mg/ml)	24	*Y* = 1.06 − 0.02*X*_1_ − 0.0003*X*_2_− 0.000005 *X*_1_*.X*_2_	−0.45	−0.39	−0.27	0.90*	*Y* = 3.46 − 0.01*X*_1_ − 0.001*X*_2_− 0.00003 *X*_1_*.X*_2_	−0.37	−0.40	−0.34	0.90*
	72	*Y* = 1.07 − 0.002*X*_1_ − 0.0004*X*_2_− 0.00005 *X*_1_*.X*_2_	−0.28	−0.28	−0.54	0.94*	*Y* = 3.23 − 0.00003*X*_1_ − 0.0003*X*_2_− 0.000007 *X*_1_*.X*_2_	−0.001	−0.05	−0.78	0.81*
	120	*Y* = 1.06 − 0.005*X*_1_ − 0.0004*X*_2_− 0.000001 *X*_1_*.X*_2_	−0.89	−0.30	0.04	0.96*	*Y* = 3.32 − 0.009*X*_1_ − 0.002*X*_2_ − 0.00001 *X*_1_*.X*_2_	−0.56	−0.53	0.066	0.80*
	168	*Y* = 1.02 − 0.003*X*_1_ − 0.0004*X*_2_− 0.000004 *X*_1_*.X*_2_	−0.89	−0.60	0.25	0.96*	*Y* = 3.20 − 0.02*X*_1_ − 0.003*X*_2_− 0.00002 *X*_1_*.X*_2_	−0.73	−0.56	0.23	0.81*
Anthocyanin content (OD/gfw)	24	*Y* = 0.18 − 0.0004*X*_1_ + 0.003*X*_2_ + 0.00002 *X*_1_*.X*_2_	−0.04	0.47	0.44	0.77*	*Y* = 0.24 + 0.003*X*_1_ + 0.0007*X*_2_− 0.000006 *X*_1_*.X*_2_	0.87	0.71	−0.41	0.90*
	72	*Y* = 0.83 + 0.01*X*_1_ − 0.005*X*_2_− 0.0001 *X*_1_*.X*_2_	0.55	1.01	−0.96	0.80*	*Y* = 0.42 + 0.002*X*_1_ + 0.0004*X*_2_ + 0.000005 *X*_1_*.X*_2_	0.48	0.37	0.21	0.86*
	120	*Y* = 0.48 + 0.001*X*_1_ − 0.0004*X*_2_− 0.000003 *X*_1_*.X*_2_	0.28	0.46	−0.18	0.46*	*Y* = 0.42 + 0.002*X*_1_ + 0.0001*X*_2_ + 0.000006 *X*_1_*.X*_2_	0.56	0.11	0.26	0.84*
	168	*Y* = 0.49 − 0.005*X*_1_ − 0.0005*X*_2_− 0.00002 *X*_1_*.X*_2_	0.79	0.33	−0.12	0.81*	*Y* = 0.37 + 0.003*X*_1_ + 0.0005*X*_2_− 0.00001 *X*_1_*.X*_2_	1.02	0.67	−0.75	0.76*
Protein content (mg/gfw)	24	*Y* = 0.76 + 0.009*X*_1_ − 0.0001*X*_2_− 0.00004 *X*_1_*.X*_2_	0.98	−0.40	−0.81	0.80*	*Y* = 0.69 − 0.003*X*_1_ − 0.0009*X*_2_− 0.000009 *X*_1_*.X*_2_	−0.70	−0.82	0.41	0.87*
	72	*Y* = 0.86 − 0.004*X*_1_ − 0.001*X*_2_ + 0.00005 *X*_1_*.X*_2_	−0.73	−0.87	0.61	0.82*	*Y* = 0.72 − 0.003*X*_1_ − 0.0007*X*_2_ + 0.000009 *X*_1_*.X*_2_	−0.71	−0.79	0.46	0.82*
	120	*Y* = 0.71 − 0.001*X*_1_ − 0.0005*X*_2_− 0.00001 *X*_1_*.X*_2_	−0.29	−0.89	0.46	0.75*	*Y* = 0.71 − 0.008*X*_1_ − 0.0006*X*_2_ + 0.000005 *X*_1_*.X*_2_	−0.92	−0.32	0.12	0.92*
	168	*Y* = 0.71 − 0.005*X*_1_ − 0.0005*X*_2_− 0.00001 *X*_1_*.X*_2_	−0.99	−0.43	0.38	0.84*	*Y* = 0.68 − 0.006*X*_1_ − 0.0008*X*_2_ + 0.000008 *X*_1_*.X*_2_	−0.90	−0.55	0.26	0.92*
Carbohydrate content (mg/gfw)	24	*Y* = 84.09 + 0.57*X*_1_ − 0.06*X*_2_− 0.0004 *X*_1_*.X*_2_	0.78	−0.36	0.12	0.87*	*Y* = 64.41 − 0.41*X*_1_ − 0.08*X*_2_ + 0.001 *X*_1_*.X*_2_	−0.87	−0.76	0.55	0.84*
	72	*Y* = 103.3 − 0.47*X*_1_ − 0.07*X*_2_− 0.0006 *X*_1_*.X*_2_	−0.84	−0.58	0.21	0.93*	*Y* = 62.6 − 0.26*X*_1_ − 0.08*X*_2_− 0.00005 *X*_1_*.X*_2_	−0.47	−0.60	−0.01	0.85*
	120	*Y* = 83.81 − 0.26*X*_1_ − 0.053*X*_2_− 0.0008 *X*_1_*.X*_2_	−0.71	−0.60	0.45	0.67*	*Y* = 65.29 − 0.37*X*_1_ − 0.10*X*_2_ + 0.0007 *X*_1_*.X*_2_	−0.63	−0.74	0.24	0.87*
	168	*Y* = 73.41 + 0.05*X*_1_ − 0.03*X*_2_− 0.0003 *X*_1_*.X*_2_	0.24	−0.51	−0.27	0.61*	*Y* = 45.37 − 0.64*X*_1_ − 0.14*X*_2_− 0.002 *X*_1_*.X*_2_	−0.88	−0.84	0.75	0.79*
MDA content (µmol/gfw)	24	*Y* = 6.15 + 0.09*X*_1_ − 0.02*X*_2_− 0.0003 *X*_1_*.X*_2_	1.02	0.75	−0.66	0.87*	*Y* = 4.80 + 0.04*X*_1_ + 0.01*X*_2_− 0.00005 *X*_1_*.X*_2_	0.58	0.59	0.03	0.94*
	72	*Y* = 6.36 − 0.008*X*_1_ − 0.02*X*_2_− 0.0006 *X*_1_*.X*_2_	0.03	0.41	0.95	0.97*	*Y* = 5.84 + 0.04*X*_1_ + 0.02*X*_2_ + 0.0001 *X*_1_*.X*_2_	0.33	0.58	0.24	0.93*
	120	*Y* = 6.26 − 0.06*X*_1_ − 0.03*X*_2_− 0.00002 *X*_1_*.X*_2_	0.27	0.30	0.57	0.97*	*Y* = 5.82 + 0.05*X*_1_ + 0.01*X*_2_ + 0.0002 *X*_1_*.X*_2_	0.40	0.51	0.26	0.94*
	168	*Y* = 9.93 − 0.16*X*_1_ − 0.03*X*_2_− 0.00002 *X*_1_*.X*_2_	0.61	0.61	0.014	0.96*	*Y* = 11.78 + 0.02*X*_1_ + 0.003*X*_2_− 0.0004 *X*_1_*.X*_2_	0.79	0.75	−0.42	0.86*
Proline content (µmol/gfw)	24	*Y* = 22.3 + 0.13*X*_1_ + 0.01*X*_2_ + 0.0007 *X*_1_*.X*_2_	0.41	0.25	0.44	0.93*	*Y* = 10.67 + 0.15*X*_1_ − 0.02*X*_2_− 0.0004 *X*_1_*.X*_2_	0.98	0.31	−0.28	0.85*
	(continued) 72	*Y* = 10.83 + 0.41*X*_1_ + 0.05*X*_2_− 0.0004 *X*_1_*.X*_2_	0.90	0.53	−0.21	0.96*	*Y* = 17.23 + 0.14*X*_1_ − 0.03*X*_2_ + 0.00002 *X*_1_*.X*_2_	0.51	0.54	0.01	0.84*
	120	*Y* = 12.48 + 0.14*X*_1_ − 0.02*X*_2_ + 0.0006 *X*_1_*.X*_2_	0.41	0.19	0.37	0.84*	*Y* = 15.7 + 0.37*X*_1_ + 0.07*X*_2_− 0.001 *X*_1_*.X*_2_	1.15	0.93	−0.89	0.95*
	168	*Y* = 12.12 + 0.37*X*_1_ + 0.06*X*_2_− 0.001 *X*_1_*.X*_2_	1.02	0.70	−0.66	0.83*	*Y* = 18.82 + 0.25*X*_1_ − 0.04*X*_2_− 0.00009 *X*_1_*.X*_2_	0.99	0.93	−0.74	0.79*
Phenolic content (mg/gfw)	24	*Y* = 0.37 + 0.06*X*_1_ + 0.0002*X*_2_− 0.00005 *X*_1_*.X*_2_	0.68	0.77	−0.43	0.81*	*Y* = 0.61 + 0.01*X*_1_ − 0.001*X*_2_− 0.000008 *X*_1_*.X*_2_	0.74	0.31	−0.08	0.79*
	72	*Y* = 0.47 + 0.008*X*_1_ + 0.001*X*_2_− 0.00005 *X*_1_*.X*_2_	0.96	0.68	−0.40	0.96*	*Y* = 0.52 − 0.001*X*_1_ − 0.002*X*_2_ + 0.0001 *X*_1_*.X*_2_	−0.04	0.23	0.86	0.97*
	120	*Y* = 0.41 + 0.01*X*_1_ + 0.001*X*_2_− 0.00005 *X*_1_*.X*_2_	0.74	0.55	−0.18	0.86*	*Y* = 0.69 + 0.01*X*_1_ + 0.003*X*_2_ + 0.00002 *X*_1_*.X*_2_	0.46	0.51	0.15	0.89*
	168	*Y* = 0.56 + 0.004*X*_1_ + 0.001*X*_2_− 0.00001 *X*_1_*.X*_2_	0.55	0.58	−0.33	0.62*	*Y* = 1.00 + 0.01*X*_1_ + 0.002*X*_2_− 0.00003 *X*_1_*.X*_2_	0.71	0.58	−0.34	0.73*
Electrolyte leakage (%age)	24	*Y* = 56.5 + 0.43*X*_1_ + 0.06*X*_2_− 0.0004 *X*_1_*.X*_2_	0.80	0.52	−0.17	0.90*	*Y* = 53.81 − 0.01*X*_1_ + 0.03*X*_2_ = 0.003 *X*_1_*.X*_2_	−0.01	0.19	0.77	0.88*
	72	*Y* = 63.07 + 0.39*X*_1_ + 0.003*X*_2_− 0.002 *X*_1_*.X*_2_	0.85	0.75	−0.74	0.72*	*Y* = 57.13 + 0.77*X*_*1+*_0.11*X*_2_− 0.00002 *X*_1_*.X*_2_	1.17	0.71	−0.68	0.94*
	120	*Y* = 81.7 + 0.20*X*_1_ + 0.04*X*_2_− 0.0009 *X*_1_*.X*_2_	0.64	0.59	−0.57	0.55*	*Y* = 84.07 + 0.14*X*_1_ + 0.05*X*_2_− 0.0008 *X*_1_*.X*_2_	0.32	0.51	−0.39	0.43*
	168	*Y* = 83.42 + 0.*X*_1_ + 0.05*X*_2_− 0.001 *X*_1_*.X*_2_	0.68	0.63	−0.63	0.58*	*Y* = 75.02 + 0.28*X*_1_ + 0.02*X*_2_− 0.0001 *X*_1_*.X*_2_	0.54	0.14	0.04	0.62*
SOD (µmole/min/mg protein)	24	*Y* = 3.84 + 0.05*X*_1_ + 0.005*X*_2_ + 0.00005 *X*_1_*.X*_2_	0.62	0.26	0.20	0.91*	*Y* = 30.10 + 0.06*X*_1_ + 0.03*X*_2_ + 0.0005 *X*_1_*.X*_2_	0.24	0.48	0.35	0.87*
	72	*Y* = 16.11 + 0.42*X*_1_ + 0.09*X*_2_− 0.0002 *X*_1_*.X*_2_	0.60	0.59	−0.06	0.87*	*Y* = 24.66 + 0.18*X*_1_ + 0.03*X*_2_− 0.0006 *X*_1_*.X*_2_	0.91	0.68	−0.60	0.77*
	120	*Y* = 30.44 − 0.07*X*_1_ + 0.04*X*_2_ + 0.0004 *X*_1_*.X*_2_	−0.22	0.56	0.24	0.65*	*Y* = 26.49 + 0.68*X*_1_ + 0.07*X*_2_ + 0.0005 *X*_1_*.X*_2_	0.70	0.32	0.10	0.92*
	168	*Y* = 26.38 + 0.52*X*_1_ + 0.03*X*_2_− 0.001 *X*_1_*.X*_2_	1.16	0.32	−0.51	0.85*	*Y* = 34.73 + 0.39*X*_1_ + 0.03*X*_2_ + 0.002 *X*_1_*.X*_2_	0.48	0.18	0.38	0.90*
CAT (µmole/min/mg protein)	24	*Y* = 0.16 − 0.01*X*_1_ + 0.0002*X*_2_− 0.00001 *X*_1_*.X*_2_	−0.49	0.25	0.88	0.74*	*Y* = 0.10 + 0.002*X*_1_ + 0.0004*X*_2_ + 0.000001 *X*_1_*.X*_2_	0.60	0.49	0.06	0.91*
	72	*Y* = 0.07 + 0.006*X*_1_ + 0.0006*X*_2_− 0.000007 *X*_1_*.X*_2_	0.95	0.44	−0.25	0.91*	*Y* = 0.08 + 0.005*X*_1_ + 0.0008*X*_2_− 0.000004 *X*_1_*.X*_2_	0.79	0.49	−0.12	0.90*
	120	*Y* = 0.16 + 0.002*X*_1_ + 0.0004*X*_2_ + 0.000004 *X*_1_*.X*_2_	0.52	0.44	0.17	0.91*	*Y* = 0.12 + 0.003*X*_1_ + 0.0005*X*_2_ + 0.00005 *X*_1_*.X*_2_	0.28	0.14	0.49	0.81*
	168	*Y* = 0.17 + 0.002*X*_1_ + 0.0003*X*_2_− 0.000003 *X*_1_*.X*_2_	0.76	0.47	−0.19	0.80*	*Y* = 0.07 + 0.001*X*_1_ + 0.003*X*_2_ + 0.000002 *X*_1_*.X*_2_	0.55	0.38	0.11	0.83*
APX (µmole/min/mg protein)	24	*Y* = 1.71 + 0.003*X*_1_ + 0.007*X*_2_− 0.0001 *X*_1_*.X*_2_	0.08	0.92	−0.66	0.79*	*Y* = 0.94 + 0.0008*X*_1_ + 0.0023*X*_2_− 0.00003 *X*_1_*.X*_2_	0.05	0.61	−0.43	0.52*
	72	*Y* = 1.54 + 0.01*X*_1_ + 0.006*X*_2_− 0.00007 *X*_1_*.X*_2_	0.55	0.96	−0.52	0.86*	*Y* = 1.08 − 0.014*X*_1_ + 0.0011*X*_2_− 0.00002 *X*_1_*.X*_2_	−0.59	0.19	−0.15	0.70*
	120	*Y* = 2.65 − 0.009*X*_1_ − 0.0004*X*_2_ + 0.0001 *X*_1_*.X*_2_	−0.41	−0.02	0.94	0.63*	*Y* = 1.30 + 0.03*X*_1_ + 0.002*X*_2_ + 0.0001 *X*_1_*.X*_2_	0.47	0.13	0.29	0.79*
	168	*Y* = 2.79 + 0.03*X*_1_ + 0.003*X*_2_− 0.00004 *X*_1_*.X*_2_	0.88	0.36	−0.21	0.84*	*Y* = 1.17 + 0.02*X*_1_ + 0.0007*X*_2_− 0.00001 *X*_1_*.X*_2_	0.89	0.10	−0.08	0.85*
GPOX (µmole/min/mg protein)	24	*Y* = 0.17 − 0.0007*X*_1_ + 0.002*X*_2_ + 0.00007 *X*_1_*.X*_2_	−0.03	0.43	0.67	0.94*	*Y* = 0.28 + 0.002*X*_1_ + 0.0006*X*_2_− 0.000002 *X*_1_*.X*_2_	0.62	0.61	−0.08	0.88*
	72	*Y* = 0.14 + 0.02*X*_1_ + 0.003*X*_2_− 0.00009 *X*_1_*.X*_2_	1.32	0.97	−1.33	0.90*	*Y* = 0.21 − 0.0004*X*_1_ + 0.0001*X*_2_ + 0.000001 *X*_1_*.X*_2_	−0.12	0.12	0.80	0.77*
	120	*Y* = 0.24 + 0.009*X*_1_ + 0.002*X*_2_− 0.00004*X*_1_*.X*_2_	1.02	1.11	−1.06	0.96*	*Y* = 0.80 + 0.02*X*_1_ + 0.0009*X*_2_− 0.00004 *X*_1_*.X*_2_	1.11	0.15	−0.35	0.87*
	168	*Y* = 0.35 + 0.007*X*_1_ + 0.001*X*_2_ + 0.000009 *X*_1_*.X*_2_	0.57	0.36	0.14	0.86*	*Y* = 1.12 + 0.01*X*_1_ + 0.001*X*_2_− 0.00004 *X*_1_*.X*_2_	1.12	0.56	−0.74	0.77*
GR (µmole/min/mg protein)	24	*Y* = 3.25-0.02*X*_1_ + 0.004*X*_2_− 0.00009 *X*_1_*.X*_2_	−0.52	0.57	0.56	0.80*	*Y* = 3.57 + 0.04*X*_1_ + 0.005*X*_2_− 0.0001 *X*_1_*.X*_2_	1.02	0.55	−0.50	0.84*
	72	*Y* = 2.68 + 0.02*X*_1_ + 0.008*X*_2_− 0.0001 *X*_1_*.X*_2_	0.55	1.02	−0.69	0.85*	*Y* = 3.23 + 0.03*X*_1_ + 0.006*X*_2_− 0.0001 *X*_1_*.X*_2_	1.08	0.84	−0.88	0.85*
	120	*Y* = 3.89 + 0.009*X*_1_ + 0.007*X*_2_− 0.0001 *X*_1_*.X*_2_	0.34	1.05	−0.80	0.85*	*Y* = 3.76 + 0.14*X*_1_ + 0.002*X*_2_− 0.0004 *X*_1_*.X*_2_	1.47	0.11	−0.95	0.86
	168	*Y* = 3.45 + 0.03*X*_1_ + 0.002*X*_2_− 0.0000008 *X*_1_*.X*_2_	0.77	0.24	−0.004	0.84*	*Y* = 3.84 + 0.05*X*_1_ + 0.005*X*_2_− 0.00008 *X*_1_*.X*_2_	0.62	0.26	0.20	0.91*
Accumulation (mg/kgfw)	24	*Y* = 17.74 + 2.21*X*_1_ + 0.42*X*_2_− 0.009 *X*_1_*.X*_2_	1.22	1.011	−1.11	0.95*	*Y* = 230.48 + 2.40*X*_1_ + 0.511*X*_2_ + 0.02 *X*_1_*.X*_2_	0.19	0.17	0.46	0.73*
	72	*Y* = 32.90 + 1.43*X*_1_ + 0.19*X*_2_− 0.005 *X*_1_*.X*_2_	1.06	0.62	−0.81	0.73*	*Y* = 348.3-1.35*X*_1_ + 0.15*X*_2_− 0.02 *X*_1_*.X*_2_	−0.13	0.06	−0.42	0.51*
	120	*Y* = 34.27 + 0.68*X*_1_ + 0.18*X*_2_− 0.004 *X*_1_*.X*_2_	0.64	0.74	−0.79	0.60*	*Y* = 19.73 + 1.53*X*_1_ + 0.18*X*_2_− 0.007 *X*_1_*.X*_2_	1.49	0.79	−1.47	0.86*
	168	*Y* = 29.43 + 0.76*X*_1_ + 0.16*X*_2_− 0.004 *X*_1_*.X*_2_	0.79	0.75	−077	0.67*	*Y* = 21.27 + 0.61*X*_1_ + 0.13*X*_2_− 0.003 *X*_1_*.X*_2_	0.84	0.80	−0.86	0.70*

On the other hand, carotenoid content in plant showed decreasing trend at all the applied concentrations of DEP, DAP and fluoride and the minimum carotenoid content was observed at combined concentration of fluoride with 300 ppm DEP as well as DAP after the exposure period of 72 hours **(**Fig. 2).

All individual treatments with either fluoride, DEP and DAP exhibited significant increase in anthocyanin pigent when compared to control plants **(**Fig. 2**)**. However, almost all binary treatments led to significantly higher anthocyanin content in comparison to respective single treatment of fluoride, DEP and DAP concentrations at all the exposure periods. Notably, combined effect of fluoride with DEP showed more anthocyanin accumulation as compared to DAP.

### Effect on soluble protein, carbohydrate and MDA content

**Figure 3:**
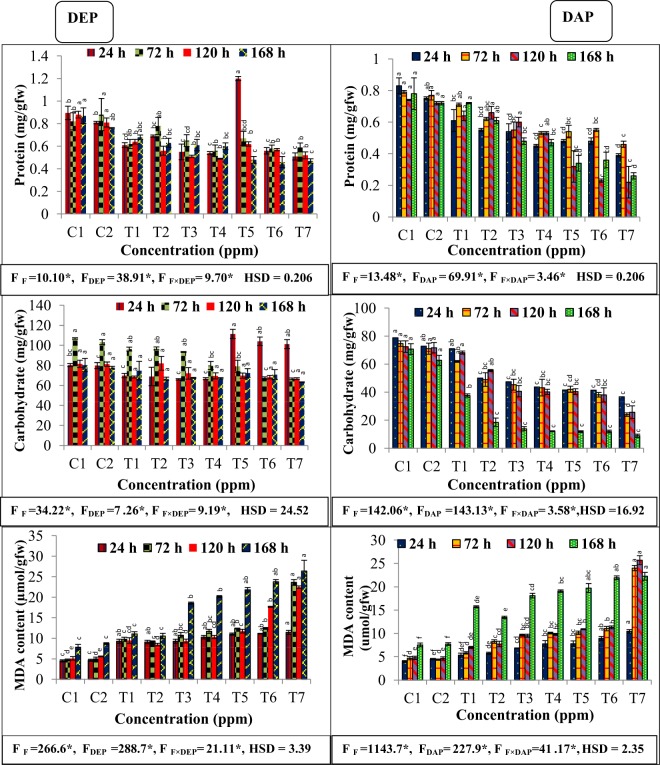
Interactive effects of fluoride and phthalates (DEP and DAP) on protein, carbohydrate and malondialdehyde (MDA) contents of *S. polyrhiza* exposed for 24, 72, 120 and 168 hours. Treatments: C1 – control, C2-control, T1 – 50 ppm F^−^, T2 –75 ppm DEP/DAP, T3- 150 ppm DEP/DAP, T4 – 300 ppm DEP/DAP, T5 – 50 ppm F^−^ + 75 ppm DEP/DAP, T6 – 50 ppm F^−^ + 150 ppm DEP/DAP, T7 – 50 ppm F^−^ + 300 ppm DEP/DAP. Data is shown as (mean ± SE, two-way ANOVA, Tukey’s HSD), * indicates significant at (*p* ≤ 0.05). Mean values with same letters signifies no significant difference between two values of different concentrations for the same exposure period (using one-way ANOVA).

All the individual treatments either with fluoride, DEP and DAP showed significant reduction (*p* ≤ 0.05) in protein content at all the exposure periods **(**Fig. 3**)**. However, binary treatment of 50 ppm of fluoride and 75 ppm of DEP exhibited high value of protein content (1.20 mg/gfw) at 24 h time period, while at other exposure periods of 72, 120 and 168 h, binary treatments of fluoride and DEP showed slight increased protein content as compared to maximum individual treatment (300 ppm) of DEP. Notably, maximum reduced protein content (0.47 mg/gfw and 0.22 mg/gfw) was found when plants were exposed to binary treatments of 50 ppm of fluoride and 300 ppm of DEP and DAP for 168 h respectively. Interestingly, combined treatment of fluoride and DAP induced marked reduction in protein content in all the combinations **(**Fig. 3**)**. Negative β-coefficients for fluoride and phthalate revealed reduction in protein content in plant, while interaction between fluoride and phthalate was positively regressed for protein content at the exposure periods of 72, 120 and 168 h **(**Table 2**)**.

Moreover, single and joint effects of fluoride, DEP and DAP also showed similar decreasing trend in carbohydrate content at all the exposure periods and presented in Fig. 3 respectively. Likewise protein content, carbohydrate content was also found to be maximum at combined treatments of fluoride and DEP (111.39 mg/gfw), 103.99 mg/gfw, 101.19 mg/gfw at 24 h time period as compared to control (T2). At other exposure periods of 72, 120 and 168 h, the significant reduction of 35, 17.8, 18.7% respectively was found at combined treatments of fluoride with 300 ppm DEP. However, exposure of plant to individual DAP and combined treatment of fluoride and DAP induced significant reduction and showed dose dependent relationship at all the exposure periods. Maximum decrease of 49.59, 66.2, 64.16 and 85.8% in carbohydrate content was found at 24, 72, 120 and 168 h respectively at highest combined treatment of fluoride and DAP. Multiple linear regression also revealed that F-, DEP and DAP treatment significantly reduced carbohydrate content, whereas binary treatment showed slight alterations (Table 2).

Single and joint effects of fluoride, DEP and DAP were also measured in terms of significant enhancement in MDA content shown in Fig. 3. Exposure of plant to individual and binary treatments provoked dose-responsive enhancement in MDA content. Maximum significant increase (*p* ≤ 0.05) in MDA content was recorded as 146.5, 374.1, 303.1 and 203.1% at binary treatments of fluoride with 300 ppm DEP whereas, combination of fluoride with highest concentration of DAP exhibited maximum enhancement of 131.7, 447.7, 453.6 and 197.9% after exposure period of 24, 72, 120 and 168 h respectively. Positive β regressions implied increment in MDA content. Interaction of fluoride with phthalate was positively regressed implying further increase in MDA content during combined stress **(**Table 2).

### Effect on total phenolic, proline content and electrolyte leakage

**Figure 4:**
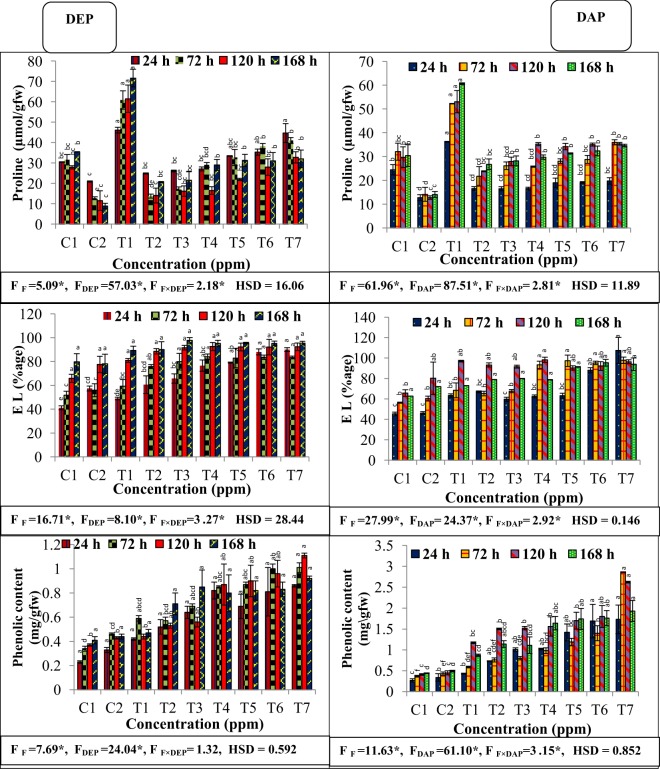
Interactive effects of fluoride and phthalates (DEP and DAP) on proline content, percentage of electrolyte leakage (EL) and total phenolic content of *S. polyrhiza* exposed for 24, 72, 120 and 168 hours. Treatments: C1 – control, C2-control, T1 – 50 ppm F^−^, T2 –75 ppm DEP/DAP, T3- 150 ppm DEP/DAP, T4 – 300 ppm DEP/DAP, T5 – 50 ppm F^−^ + 75 ppm DEP/DAP, T6 – 50 ppm F^−^ + 150 ppm DEP/DAP, T7 – 50 ppm F^−^ + 300 ppm DEP/DAP. Data is shown as (mean ± SE, two-way ANOVA, Tukey’s HSD), * indicates significant at (*p* ≤ 0.05). Mean values with same letters signifies no significant difference between two values of different concentrations for the same exposure period (using one-way ANOVA).

Present investigation revealed that individual treatment of fluoride, DEP and DAP led to significant increase in phenolic content, when plants of *S. polyrhiza* were exposed for 24, 72, 120 and 168 h time period **(**Fig. 4**)**. However, content was maximum in combination of fluoride with highest concentration of either DEP or DAP as compared to control. Maximum percent increase was recorded as 160.6, 119.5, 158.1 and 109.1% at the highest binary treatment of fluoride with DEP, while combined treatment with DAP showed 408.3, 565.1, 482.2 and 293.8% at respective time periods of 24, 72, 120 and 168 h.

As for the proline content, individual treatments with both fluoride, DEP and DAP led to significant accumulation of proline in fronds of *S. polyrhiza*, with maximum accumulation found at 50 ppm fluoride **(**Fig. 4**)**. However, combined treatments of fluoride either with DEP and DAP showed maximum proline content of 44.66, 41.02, 32.79, 32.06 µmol/gfw and 19.78, 35.97, 35.39, 34.63 µmol/gfw as compared to their respective controls at high combination of fluoride with 300 ppm DEP and DAP after 24, 72, 120 and 168 h respectively.

Present observation also revealed increased electrolyte leakage during individual as well as combined stress of fluoride, DEP and DAP. However, binary treatments showed more enhanced leakage of electrolytes as compared to individual treatment of fluoride, DEP and DAP. Maximum leakage of 90.02 and 107.36% was recorded at binary treatment of fluoride with 300 ppm DEP and DAP at the time period of 24 h, while at other exposure periods, percentage of leakage increased moderately and become almost constant with increasing combined treatments. Positive β egression depicted individual effect of fluoride and phthalate on increased content of phenolic, proline content and leakage of ions at all the exposure periods (Table 2).

### Effect on enzymatic activities

**Figure 5:**
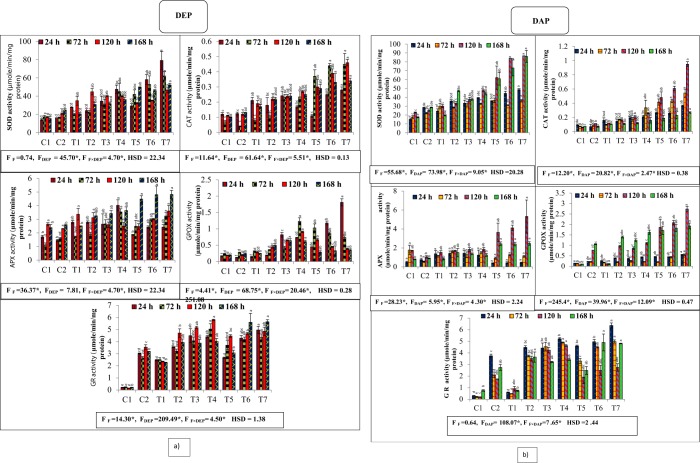
Interactive effects of fluoride and phthalates (DEP and DAP) on specific activities of antioxidative enzymes (SOD, CAT, APX, GPOX and GR) of *S. polyrhiza* exposed for 24, 72, 120 and 168 hours. Treatments: C1 – control, C2-control, T1 – 50 ppm F^−^, T2 –75 ppm DEP/DAP, T3- 150 ppm DEP/DAP, T4 – 300 ppm DEP/DAP, T5 – 50 ppm F^−^ + 75 ppm DEP/DAP, T6 – 50 ppm F^−^ + 150 ppm DEP/DAP, T7 – 50 ppm F^−^ + 300 ppm DEP/DAP. Data is shown as (mean ± SE, two-way ANOVA, Tukey’s HSD), * indicates significant at (*p* ≤ 0.05). Mean values with same letters signifies no significant difference between two values of different concentrations for the same exposure period (using one-way ANOVA).

All applied treatments to experimental plants exhibited significantly higher SOD activity when compared to control, which was mainly pronounced in combined treatments of fluoride with DEP and DAP during all the durations **(**Fig. 5**)**. Highest SOD activity was recorded at the binary treatment of fluoride with 300 ppm DEP after 24 h time period in comparison to individual treatments. With increasing time interval, reduced activity was observed in binary treatments. However, the treatment of fluoride with highest concentration of DAP showed maximum SOD activity after the time interval of 120 and 168 hours as compared to single treatments.

All individual treatments significantly elevated CAT activity in exposed fronds as compared to control during all the exposure periods, although all binary treatments triggered even higher CAT activity. Maximum CAT activity was observed at the highest combination of fluoride with DEP as well as DAP during the time interval of 24, 72 and 120 h, while after the exposure period of 168 h, reduction in CAT activity was found.

In the present investigation, APX activity showed significant increase with increasing dose and duration. Maximum activity was observed when binary treatments of fluoride with 300 ppm DEP were given to *S. polyrhiza* after the time period of 168 h, while combined exposure of fluoride with 300 ppm DAP showed highest APX activity after 120 h time period. However, binary treatments induced reduced CAT activity after 24 and 72 h time period when compared to control and individual treatments of DAP.

In *S. polyrhiza*, enhanced GPOX activity was measured upon exposure to individual fluoride, DEP and DAP concentrations and their combinations comparison to control. Binary treatment of fluoride with DEP showed reduced GPX activity as compared to individual treatment after 24 hours and then become maximum at the highest combination of fluoride with 300 ppm DEP. While, reduced GPX activity was observed at same combined treatment after 72, 120 and 168 h. However, individual treatment of DAP as well as combined treatment of DAP with fluoride showed increasing trend at all the durations and maximum activity was found after the time period of 120 h.

In this observation, all individual treatments induced significant increase in GR activity, while binary treatment of fluoride with 75 ppm DEP or DAP showed decreased GR activity when compared to control during all the durations. However, maximum increase in the activity was found at highest combined concentration of fluoride with 300 ppm DEP after 168 h, while those treated with combination of fluoride with 300 ppm DAP showed higher GR activity after the exposure period of 24 h.

### Scanning electron microscopic studies

**Figure 6:**
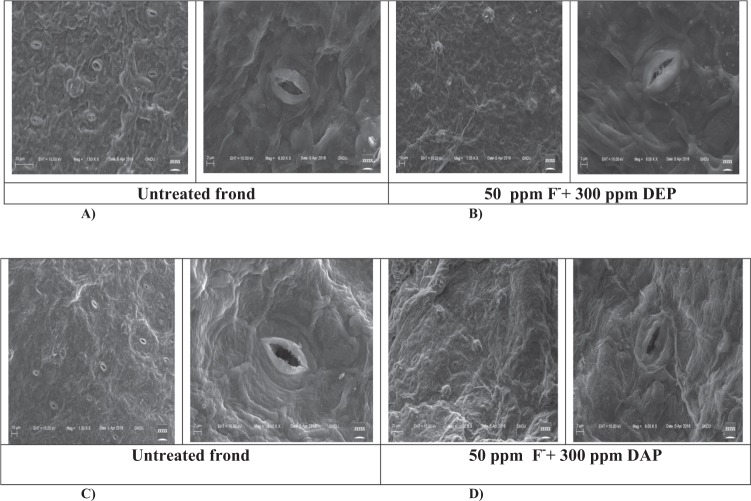
Scanning electron micrographs depicting (**A**) open stomata (untreated) (**B**) closed stomata (treated) fronds of *S. polyrhiza*.

Scanning electron micrographs revealed marked influence of treatments on stomatal movements and morphology of stressed fronds of *S. polyrhiza* over unstressed one. Stomata of control fronds were open, while stomata of treated fronds were mostly closed. Various other cellular changes like deformed cell shapes and collapsed cells were prominent in treated fronds as compared to untreated fronds are viewed through SEM imaging **(**Fig. 6).

### Confocal laser scanning electron microscopic studies

**Figure 7:**
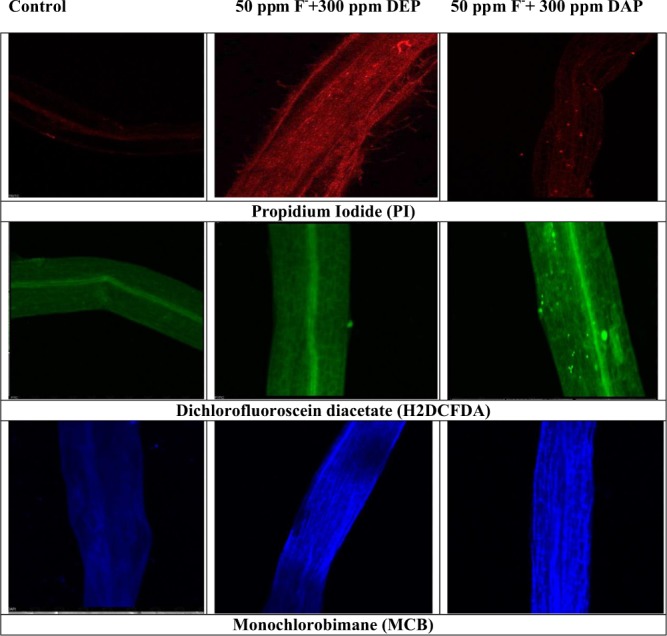
Confocal micrographs of roots of *S. polyrhiza* showing interactive effects of fluoride with DEP and DAP.

Roots of stressed plants were treated with different fluorescent dyes. In present investigation, PI treated roots showed more red fluorescence, indicating dead cells and disruption in membrane integrity as compared to control that showed less or no fluorescence. Green fluorescence emission of DCFDA dye treated roots of plant confirmed the accumulation of reactive oxygen species during oxidative stress, whereas blue fluorescence emission of MCB dye treated roots revealed enhanced GSH levels (Fig. 7**)** over control roots.

## Discussion

Plants play a pivotal role as a bioindicator to give us insights into cumulative and interactive effects of environmental contaminants. Deleterious effects on them give us alarming signals for protecting our environment and human beings. In our study, we reported the effects of binary concentrations of fluoride and phthalates on accumulation, growth and biochemical parameters of *S. polyrhiza*. The potential of aquatic plants for uptake of organic compounds and removal of nutrients from wastewater has been vastly explored^16,17^. The present investigation on *S. polyrhiza* showed that DEP, DAP and fluoride tend to accumulate in fronds of an aquatic macrophyte, which is in accordance with the data reported for other aquatic as well as terrestrial plants^18,19^. As illustrated in Table 1, accumulation of DEP and DAP in the plants decreased with the increase in the exposure periods. Higher DEP concentrations in growth medium led to enhanced DEP uptake which then gradually decreased with increasing exposure time, while uptake of DAP decreased with increasing concentrations though the accumulated content was much higher than DEP. Although majority of studies reported the individual effects of fluoride and phthalates on various plant species, but there is no report about the fluoride-phthalate interaction mechanism in any plant species. Since, the laboratory experiments are performed under controlled conditions, so the results obtained partly give us an idea about the consequences under environmental conditions, which are much more complex and unstable. From these results, it was revealed that fluoride and phthalates considerably influenced each other uptake, when present together in the nutrient medium. In fronds exposed to binary treatments of fluoride with DEP, higher fluoride content was observed as compared to accumulated DEP content, while binary treatment with DAP, higher DAP uptake was observed, which indicated that fluoride stimulated DAP uptake. This suggested synergistic relationship existing between fluoride and phthalates. However, the effect of fluoride on alleviation of DEP toxicity might be attributable partly to the fluoride induced decreased accumulation of DEP in fronds. Different responses in accumulation and interaction of fluoride with DEP and DAP attributed to genotype of plant, contaminant, pH of the medium, temperature and humidity^21,22^. Interestingly, uptake of organic compounds by this free floating macrophyte is driven by simple process of diffusion and then penetration into a leaf as solutions^23,24^. Since, plants do not have any specific transporters for these organic compounds, so their movements into the plants mainly depends on their physicochemical properties such as hydrophobicity, polarity, aqueous solubility and molecular weight of the organic contaminant^24,25^. Our results revealed that longer exposure periods resulted in less accumulation of phthalates in plants, which might be due to metabolic pathways for the biotransformation of organic contaminants adopted by the plant. Exposure of aquatic plants to organic compounds resulted in i) fast uptake or sequestration of the compound into vacuoles ii) Transformation or degradation of the compound *via* volatilization, lignification or metabolization to carbon dioxide and water iii) Assimilation into plant tissues as non-toxic compounds^24,26^.

In our experiment, we found that accumulation of F, DEP and DAP in fronds of an aquatic macrophyte was accompanied by considerable cellular and metabolic changes, ultimately enhancing tolerance ability of plant. One of the visible change was growth inhibition of plant due to toxic effects of added F, DEP and DAP in the medium after all the exposure periods. Present study demonstrated duration-dependent response of plant against binary as well as individual treatments stress on growth parameters of plant. Noticeably, maximum effect was observed after longer duration combined treatments of fluoride with DEP or DAP. Reduction in growth and dry to fresh weight ratio under toxic conditions might be due to disruption in metabolic pathways and adverse effect on cell division, cellular growth or enhanced accumulation rate of both fluoride and phthalate in combined treatments^27^. Also, there may be decreased transport of nutrients from the nutrient medium to the plant which caused growth retardation. Results are in coherence with studies that reported the effects of fluoride and phthalates on biomass reduction in *S. polyrhiza*^28,29^. Combined effects of fluoride and phthalates further inhibited plant growth compared to single treatment of F^−^, DEP and DAP.

A reduction in total chlorophyll and carotenoid content was observed in both individual and combined treatments of F^−^, DEP and DAP during all the exposure periods. Influence of applied treatments of DEP and DAP on photosynthetic pigments is a consequence of nutrient deficiency such as iron or altered activity of δ-ALA-D (delta-aminolevulunic acid dehydratase) that induced inhibition of chlorophyll biosynthesis^30^. Moreover, another reason behind the reduction of photosynthetic pigments may be due to lipid peroxidation in the membrane of chloroplasts^31^. Perusal of literature reported reduction in photosynthetic content in various aquatic plants exposed to stress such as *Lemna gibba* and *Lemna minor*^32,33^. However, fronds exposed to DAP showed highest amount of total chlorophyll content during 168 h time period as compared to other exposure periods, suggesting that individual as well combined stress induced disruption or disorganization of thylakoid membranes and activation of chlrophyllase enzyme led to enhanced pigment content^34^. Moreover, we observed a significant decrease in carotenoids content at combined stress of F with DEP as well as DAP, implying their role in inducing inhibition of biosynthesis of carotenoids. As imposed treatments with both DEP and DAP did not induce much reduction in carotenoid content as compared to control, suggesting minimal effect on carotenoids. Similar reports are there which revealed the toxic effects of anthracene (PAH) on photosynthetic pigments of *Lemna gibba*^35^. In addition, anthocyanin pigment content significantly enhanced in this investigation, implying stress activated the gene which stimulated anthocyanin biosynthesis. Though, the effect of DEP on anthocyanin pigment was negligible as compared to DAP in this observation. Increased content is a part of strategy adopted by the plant to minimize the deleterious impacts of stress.

Similarly, evaluation of total soluble protein content revealed significant toxic effects of F^−^, DEP and DAP treatments on aquatic plant, which is an important parameter for determining phytotoxic effects of environmental contaminants. Highest reduction in protein content was recorded in fronds when exposed to combined concentration of fluoride and phthalates. Overall, DEP and DAP are toxic and significantly decreased protein content of *S. polyrhiza*, probably due to increased protease activity or other catabolic enzymes. Also, decreased level of protein content in response to fluoride and phthalates could be explained by the fact that toxicant formed during the combination of fluoride and phthalate might altered cytochrome oxidase activity and various respiratory pathways^36^. These results are consistent with earlier findings that both fluoride and phthalate provoked oxidative stress and lead to protein degradation in plant species^28^. Slight increase of protein content at some durations attributed to the alteration in protease activity to cope up with the stress. Moreover, in response to the applied treatments, carbohydrate showed decreasing trend which might help in the adjustment of osmotic imbalance by the conversion of starch to sugar in the presence of hydrolytic enzymes or to supply more ATP to the stressed cells *via* increasing respiration rate^37–39^. Although the individual as well as combined toxic effect of DAP on carbohydrate content was much more pronounced as compared to DEP at all the concentrations and exposure periods, implying toxic behaviour of DAP on plant.

On the other hand, single and combined effects of fluoride, DEP and DAP, measured in terms of formation of MDA content, byproduct of lipid peroxidation of plasma membrane, found to be increased in the present study, since, it is well documented that both fluoride and phthalate triggered oxidative stress, that damages cell membrane and consequently enhanced MDA content. Furthermore, this result is supported by the findings of Ting-Ting *et al*.^40^ and Cai *et al*.^20^ that reported increased MDA content in mung beans under phthalates (DBP and DEHP) stress and tea plant under fluoride stress respectively. Higher phthalate concentrations can induce oxidative stress, which has already been reported for several plant species.The mechanism behind the formation of reactive oxygen species (ROS) is a consequence of interaction of the contaminants with the lipid rich membrane, subsequently led to conformational changes in the membrane by the activation of NADPH-oxidase located there, hence generating ROS^41^.

To gain further insights into the osmotic adjustment potential of the plant, we evaluated proline content of the plant. Our data revealed that the accumulation of proline enhanced significantly during all the treatments, whilst plant tends to accumulate maximum proline contents at individual fluoride concentrations. Although, combined concentrations of fluoride with DAP and DEP showed more proline accumulation as compared to individual concentrations in our study. Being an osmoprotectant, proline contributes in the detoxification of ROS and protects integrity of membrane^42^. Enhanced proline accumulation is an indicator of stress in *S. polyrhiza*. This also confirmed a dynamic bond between increased lipid peroxidation and proline content in this observation. Our results are in coherence with earlier findings of impact of fluoride and phthalates on proline content in plants^40,43^. Additionally, we detected disruption in the membrane integrity in the form of ion leakage and found that to be maximum at combined concentrations of fluoride and phthalates. These results correlated well with the increased lipid peroxidation and leakage of ions during combined stress, suggesting additional evidence that expression of proline and MDA is important during severity of combined stress, as they play a crucial role in protecting cellular membrane from disruption, consequently counteract the effects of stress in *S. polyrhiza*. Apart from this, increased accumulation of phenolic content in the fronds attributed to the activation of hexose-monophosphate pathway along with the release of bound phenols in stressed fronds^44^.

Generally, reactive oxygen species (ROS) are inescapable byproducts of metabolic processes such as photosynthesis and respiration in plants^45,46^. However, environmental contaminants such as heavy metals, herbicides, pesticides and other xenobiotics pose stress in plants and overwhelmingly generate ROS and free radicals, consequently compromising various metabolic and cellular activities. Meanwhile, plants possess an armory of antioxidant enzymes to scavenge ROS and free radicals and to counteract the deleterious effects. While, activity of antioxidant enzymes varies, since it depends on plant species, concentration, and exposure duration of contaminants affecting the plant^42,47^. Activation of enzymatic activities (SOD, CAT, APX, GPOX and GR) play a crucial role in detoxification and thus, represent altered redox status of the cell under stress^8^.

Among them, SOD is an essential enzyme of antioxidant defense system, catalyses the diminution of superoxide radicals to molecular oxygen and hydrogen peroxide at a very high pace^48^. In our present investigation of evaluation of enzymes activity, we observed that the SOD activity in fronds under F^−^, DEP and DAP enhanced, with the maximum elevation found at combined stress. Surprisingly, SOD activity was recorded in plants under DAP stress enhanced significantly with increasing concentration and exposure period, while SOD activity under DEP stress reduces initially and then increases after longer exposure periods, though showed increasing trend with the proceeding concentrations. Probable reason behind increased SOD activity is attributable to the adverse effects of combined concentrations mediated formation of enormous superoxide radicals. Ting-Ting *et al*.^40^ stated that, exposure to higher concentrations of DBP and DEHP also resulted in significant rise in SOD activity in mung bean seedlings. Karmakar *et al*.^49^ also reported increased SOD activity in aquatic macrophytes under fluoride stress. Subsequent increase in the activity reflected that SOD enzyme was stimulated by the formation of superoxide radicals, to protect the plant from the toxicity of fluoride and phthalates.

Further, removal of hydrogen peroxide involves the activation of catalase enzyme (CAT) from antioxidant machinery. CAT activity showed increased activity with the highest activity was recorded at maximum combined concentrations of fluoride with DEP as well as DAP. Although the present investigation revealed increased CAT activity with progressive increase in dose and duration, but the activity reduced at the highest exposure period as compared to the CAT activity found after other initial exposure periods, suggesting that scavenging potential of this enzyme declined in plants exposed to stress for longer duration. Thus, it is obvious that combined stress - induced damage might have increased at high concentrations and durations. Also imbalance in dynamic equilibrium existing between ROS population and pace of elimination or scavenging *via* enzymes resulted in deleterious effects on plants. Results are in agreement with the previous reports of the authors who reported decreased CAT activity in plants under phthalate and fluoride stres^49,50^.

Despite of the distinct antioxidative enzymes involved in scavenging of ROS, POD also plays a chief role in the breakdown of hydrogen peroxide. APX is one of the types of POD that uses ascorbate as an electron donor to eliminate hydrogen peroxide, which is the first step in ascorbate-glutathione cycle^48,51^. Importantly, to cope up with oxidative stress, activities of SOD and APX need not to be required sufficiently high, but balance in the equilibrium existing between SOD and APX is needed^45^. Similar trend was displayed by APX as by SOD in this observation. Compared to control, a dose-duration dependent maximum increase by 88.2% at 168 h under maximum concentrations of fluoride with DEP, while combined treatment with DAP showed maximum activity at 120 h. A possible explanation underlying enhanced APX activity was probably increased level of peroxides in the chloroplast of plant and stimulation of APX activity provides resistance to stress. High response of APX to phthalate in plant was also well demonstrated by Liao *et al*.^51^. Authors also suggested the defensive role of APX exposed to stress by its striking role to remove peroxides^48^.

Meanwhile, GPOX another type of POD, exhibited increased activity initially under combined exposure of fluoride with DEP exposure after 24 h and then reduced drastically at other exposure periods. Contrary to this, combined treatments of fluoride with DAP showed enhanced GPX activity in concentration-duration dependent manner. Interestingly, plant showed its efficiency to up-regulate both types of peroxidases to counteract the effects of peroxides generated under stressful environments.

Likewise APX, GR is also one of the main components of ascorbate-glutathione cycle which is generally located in the chloroplast, but also found in mitochondria and cytosol^48,52^. GR plays an important role in maintaining reduced/oxidized glutathione (GSH/GSSG) ratio by catalysing the reduction of GSSG to GSH and thus assured efficient recovery of glutathione during oxidative damage^1,53^. Present observation showed that GR activity has increased with enhanced dosage of fluoride, DEP and DAP individually as well as combined, implying activation of ascorbate-glutathione cycle at a very high pace to scavenge ROS generated in chloroplast of stressed fronds. Since, maintenance of glutathione pool is essential so that it does not intervene in phytochelatin biosynthesis. Basically, phytochelatins play a critical role in sequesteration of various environmental contaminants and inactivate them *via* conjugates formation^54,55^. Hence, a crucial role is played by GR in the protection of plant chloroplasts. Our results are consistent with the earlier reports on the increased GR activity in plants under phthalate exposure^56,57^.

**Figure 8:**
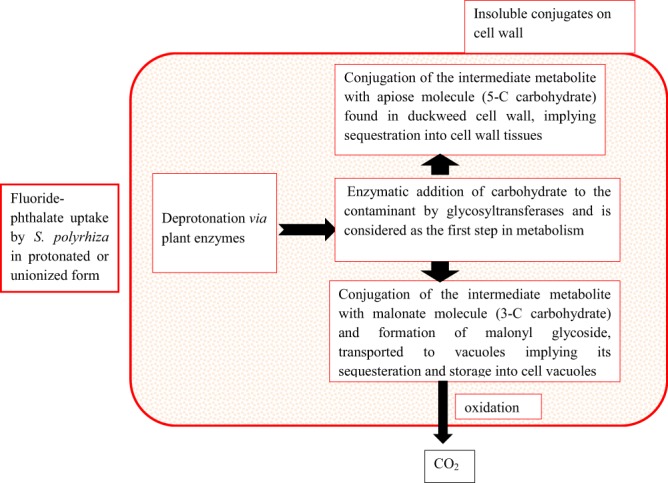
Hypothetic schematic depiction of step-wise detoxification mechanism of fluoride-phthalate by *S. polyrhiza* (Modified from Kvesitadze *et al*.^78^.

Oxidative stress faced by *S. polyrhiza* under fluoride and phthalates was confirmed by viewing electron micrographs of adaxial surface of stressed fronds which showed many closed stomata as compared to unstressed fronds **(**Fig. 6**)**. *S. polyrhiza* attained DEP stress tolerance attributes by showing alterations in stomatal movements. Furthermore, our results are supported by confocal micrographs which depicted no or less fluorescence in the roots of control plants, while high fluorescence was observed in treated roots implying that *S. polyrhiza* accumulated reactive oxygen species and fluorescence helps in determining apoptosis **(**Fig. 7**)**. A hypothetical schematic representation depicting detoxification strategy adopted by *S. polyrhiza* is presented in Fig. 8.

### Conclusions and future prospectives

In this investigation, fluoride and phthalates influenced the uptake of each other and revealed synergistic and/or antagonistic effects during combined treatments. The greatest impact of fluoride- phthalates induced stress was recorded at the highest concentration of combined treatments. Analysis showed that individual treatment of fluoride and phthalates induced oxidative damage to some extent but combined treatment further triggered oxidative burst and was more prominent in initial exposure periods, which then alleviated with increase in exposure periods. This indicates that this aquatic plant may adapt to the combined stress with due course of time by activating antioxidant machinery, thus maintaining redox imbalance. It may be possible that these contaminants (Fluoride-phthalates) bind to the plant cell wall and are metabolized or sequestered by plant in the vacuoles and converted into non-toxic form. Higher DAP uptake mediated by fluoride in combined treatments led to more ecotoxicological effects on plant as compared to DEP. Delineation of fate of fluoride-phthalates within plant tissues is required for better understanding of the mechanisms of toxicity and its sequesteration within plant cells.

## Materials and Methods

### Experimental plant material and treatments

*Spirodela polyrhiza* L. Schleiden (Duckweed) collected from Sewage Treatment Plant (STP) of Guru Nanak Dev University, Amritsar, India and cleaned properly with distilled water. Plants were acclimatised for a week in 3% Hoagland’s nutrient medium in a growth chamber illuminated with cool white fluorescent with light intensity (115 μmol m^2^ s^−1^) and photoperiod of 16 hours light and 8 hours dark period at 25 ± 2 °C (OECD, 2006). After acclimatization, healthy plants were exposed to different concentrations of fluoride and phthalates (DEP or DAP). Stock solutions (100 ppm) of (NaF) was prepared in Hoagland’s nutrient medium. Stock solution (500 ppm) of phthalates was prepared using 1 ml of ethanol and 2–3 drops of Tween-20 and distilled water in required proportion to obtain the solubility of solution^58^. Further required concentrations for treatment were prepared by diluting stock solutions of phthalates in Hoagland’s nutrient medium. Treatments of fluoride and phthalates (DAP and DEP) were applied to desired concentrations either individually as well as in binary combinations, to make the following treatments (1) Control (Hogland’s medium); (2) Control (Hogland’s medium + Tween 20 + ethanol); (3) 50 ppm F^−^; (3) 75 ppm DEP or DAP; (4) 150 ppm DEP or DAP (5) 300 ppm DEP or DAP (6) 50 ppm F^−^ + 75 ppm DEP or DAP (6) 50 ppm F^−^150 ppm DEP or DAP (6) 50 ppm F^−^ + 300 ppm DEP or DAP. These concentrations were decided from pre-experimental studies, in which various lower and higher concentration of fluoride and phthalates were used. Fluoride showed maximum damage to the plant growth at 50 ppm concentration, while EC_50_ of both the pthalates was calculated as 229.08 ppm (DAP) and 271.60 ppm (DEP).

Diethyl phthalate (99.0% purity, CAS: 84-66-2) and diallyl phthalate (97.0% purity, CAS: 131-17-9) were purchased from Hi-Media, Mumbai (India). Other chemicals used as ingredients of Hoagland’s nutrient medium (3%) were of analytical grade.

Harvesting of plant material was done after 24, 72, 120 and 168 hours. Plant material was thoroughly washed, blotted to remove the excess water and then stored in deep freezer (−80 °C). Plant material was then assayed for various growth and biochemical parameters.

### Analysis of accumulated phthalate content in *S. polyrhiza* using reverse phase high performance liquid chromatography (RP-HPLC)

From plant samples, phthalates were extracted by using the ultrasonication method of Ma *et al*.^59^ with slight modifications. 500 mg of plant sample was extracted with 20 ml acetone followed by ultrasonication of 30 min. Hexane (20 ml) was added for further extraction and again sonication was done for 30–35 min. The extract obtained was filtered and evaporated to 1 ml at 40 °C in rotary evaporator. Acetonitrile (4 ml) was added to remaining extract to make up 5 ml volume, filtered using 0.22 µm syringe filter and then analysed using reverse phase high performance liquid chromatography (RP-HPLC).

### Chromatographic conditions

Phthalates in our present study were analysed using Shimadzu reverse phase high performance liquid chromatography coupled with PDA detector (RP-HPLC-PDA) Nexera system (Shimadzu, Japan). The system was equipped with degasser, autosampler, solvent mixing unit, column oven, PDA detector and CBM-20A (system controller). Chromatographic column used for separation of phthalates was C_18_ having dimensions of 150 × 4.6 mm with pore size of 5 µm. The mobile phase used for separation were acetonitrile (Solvent A) and HPLC grade water (Solvent B). The gradient elution system was initiated with 50% A and 50% B, followed by 40% B for 2.00 min., 30% B held for 4.00 min, then 35% B for 5.00 min, followed by 35% B for 8.00 min and ended at 20.01 min. Flow rate was kept at 0.85 ml/min and injection volume was 10 μL. The maximum pressure limit of the instrument was 15000 psi. Column temperature was 38 °C and wavelength was set at 232 nm. Phthalates in the samples were detected on the basis of comparing retention time of standard peaks at absorbance of 232 nm (Supplementary File S1).

### Method validation

A method was developed and validated for the estimation of phthalates using reverse phase-high performance liquid chromatography (RP-HPLC). The stock solution (500 mg/l) of DAP and DEP was prepared in HPLC grade acetonitrile and working solutions (5–400 mg/l) were prepared through serial dilution of stock.

The method validation was carried out using normalized guidelines of International Conference on Harmonisation of Technical Requirement for Registration of Pharmaceutical for Human Use^60^. Firstly, the calibration curve was obtained using peak area versus concentration of each phthalate. The analyzed validation parameters are precision, accuracy, limit of detection (LOD) and limit of quantification (LOQ). The precision is repeatability of a system which is expressed as percentage of relative standard deviation (% RSD) and was calculated from six replicates of phthalate. Whereas, accuracy is the proximity between the conventional value and the observed value and expressed as percentage recovery. The determination of LOD and LOQ was done on the basis of signal-to-noise ratio (S/N) which is 3.3 and 10 respectively^60^.

### Analysis of accumulated fluoride content in *S. polyrhiza* using ion chromatography

Fluoride content accumulated by plant was measured by using the protocol of Zhou *et al*.^61^ with slight modifications. Plant samples were oven dried at 105 °C for first 25 min and then dried at 80 °C for next 48 hours. About 1 g of oven dried plant sample was taken in crucible and pulverised properly. The plant material was further soaked in 3 ml of 10% Mg(NO_3_)_2_ and 1 ml of 10% NaOH for 30 min. The samples were then evaporated and incubated at 200 °C in an oven. After carbonisation, samples were ashed in muffle furnace for 6 hours at 550 °C. Crucibles containing ashed material was finally cooled and rinsed with millipore water. Rinses of these samples were combined and total volume was made upto 30 ml. Fluoride content in plant samples was then analysed using ion chromatography (Metrohm Ion Chromatography, Orion-960) with known concentration of fluoride solution for calibration^28^.

### Determination of growth parameters

For determining fresh weight, plant material was washed several times, dried on filter paper to remove excess water and then weighed. Plants were oven dried at 105 °C for first 20 min and then dried at 80 °C for two days to obtain constant dry weight. Percentage change in fresh weight and dry weight to fresh weight ratio was calculated.

### Determination of photosynthetic pigments

Chlorophyll content was extracted according to protocol given by Arnon^62^. Plant material was crushed in acetone (80%) and the extract was centrifuged for 10 min. Absorbance of chl a and chl b was taken at 663 and 645 nm respectively using ELISA plate reader and concentrations were expressed in µg/ml. Carotenoid content was measured by the method of Lichtenthaler and Wellburn^63^. Absorbance of the extract was observed at 470 nm and the concentration was expressed in µg/ml. Anthocyanin content was assayed by the protocol given by Mancinelli^64^. 1 g of fresh plant material was crushed in 3 ml of acidified methanol (99 ml methanol: 1 ml HCl). Extract was centrifuged for 20 min at 12000 rpm at 4 °C. Supernatant was collected and incubated at 4 °C for 24 h. Absorbance was taken at 530 and 657 nm. Results were expressed as OD/gfw.

### Determination of soluble protein, carbohydrate and MDA content

The measurement of soluble protein content was done by homogenising the plant material in potassium phosphate buffer (pH = 7). Extract was centrifuged and protein content was determined according Bradford method^65^, where bovine serum albumin was used as a standard. Lipid peroxidation was determined by measuring malondialdehyde (MDA) content^66^. Homogenisation of plant material was done in 0.1% trichloroacetic acid (TCA) and centrifuged for 10 min. Reaction mixture containing supernatant, 0.5% thiobarbituric acid (TBA) and 20% TCA was heated at 95 °C in a boiling water bath for 30 min and then rapidly cooled on ice. After centrifugation at 10,000 rpm for 10 min, the absorbance of the supernatant was read at 532 nm and non-specific absorption was corrected by subtracting the absorbance value observed at 600 nm. The concentration was expressed as µmol/g fresh weight. For carbohydrate determination, anthrone method was used^67^. Glucose was used as a standard.

### Estimation of antioxidant enzymatic activities

Frozen plant material (1 g) was ground in liquid nitrogen and homogenized in 3 ml of 0.1 M ice cold potassium phosphate buffer (pH = 7) using chilled pestle mortar. Homogenate was then centrifuged at 12000 rpm for 20 min at 4 °C. Supernatant was collected and used for the analysis of enzymatic activities. Superoxide dismutase assay (SOD) (EC 1.15.1.1) was done by the method given by Kono *et al*.^68^ Catalase assay (CAT) (EC 1.11.1.6) was performed by the method given by Aebi^50^. Ascorbate peroxidase assay (APX) (EC 1.11.1.11) was done by following the protocol of Nakano and Asada^69^. Guaiacol peroxidase assay (POD) (EC 1.11.1.7) was done as per the method given by Putter^70^. Activity of glutathione reductase (GR) (EC. 1.6.4.2) was assayed by the protocol given by Carlberg and Mannervirk^71^.

### Determination of total phenolic, proline content and electrolyte leakage

Total phenolic content was determined according to a procedure given by Singleton and Rossi^72^. Results were expressed as mg/gfw. Proline content in plant was determined by the method given by Bates *et al*.^73^. Results were expressed as µmol/g tissue.

Electrolyte leakage was determined as per the method given by Dionisio-sese and Tobita^74^. Fronds of *S. polyrhiza* (0.2 g) were cut into small pieces and added in the test tubes filled with distilled water (10 ml). Tubes were incubated at 40 °C for 2 h. After cooling, initial electrical conductivity (EC1) was measured using conductivity meter. Then samples were autoclaved at 121 °C for 20 min to release all the electrolytes. Samples were then cooled to room temperature and final electrical conductivity was recorded (EC2). Percent electrolyte leakage was calculated using following equation:

$${\rm{Electrolyte}}\,{\rm{leakage}}( \% )=\frac{{\rm{EC}}1}{{\rm{EC}}2}\times 100$$

### Scanning electron microscopy (SEM)

Scanning electron microscopy was performed to detect the stomatal response under stressful conditions. The stomatal response of control and treated fronds of *S. polyrhiza* was observed by using scanning electron microscope (Carl Zeiss-EvoLS 10). For sample preparation, leaf samples were fixed overnight in 2.5% glutaraldehyde prepared in 0.1 M potassium phosphate buffer (PPB). The leaf samples were washed with distilled water and then further dehydrated in different ethanol series (30%, 50%, 70% and 90%) for 15 min individually. Adaxial surface of leaves were then placed on metal stubs The surface features of fronds were observed under the scanning electron microscope at a voltage of 15 KV and stomata were viewed under the resolution of 500–4000 nm.

### Confocal laser scanning microscopic (CLSM) studies

For confocal microscopy, roots of control and treated plant samples were washed with distilled water and then treated with different fluorescent dyes i.e. Propidium iodide (PI), dichlorofluorescein diacetate (H2DCFDA) and monochlorobimane (MCB) respectively in dark for 10 min separately for the study of cell viability, detection of ROS and GSH levels occurred during stress in plants. A 50 μM solution of PI was prepared according to the protocol of Gutierrez-Alcala, *et al*.^75^ Detection of ROS levels in the roots of *S. polyrhiza* was done by following the method given by Ortega-Villasante *et al*.^76^ Localization of glutathione (GSH) in roots of *S. polyrhiza* was done by the method of Fricker and Meyer^77^. For sample preparation, roots were washed and placed on glass slide over a drop of water to prevent dehydration and then covered with cover slip to visualise under confocal laser microscope. For propidium iodide (PI), He-Ne gas laser was used to excite the electrons at wavelength of 535 nm, multiline argon gas laser was used for 2, 7 dichloroflurescein (DCF) to excite the electrons at the wavelength of 488 nm and for monochlorobimane (MCB) excitation wavelength of 380 nm was used.

### Statistical analysis

All the experimentation was performed in triplicates and expressed as the mean ± standard error. The data was subjected to two-way analysis of variance (ANOVA) for assessing the effect of fluoride and phthalates on *S. polyrhiza*. Analysis of all the experiments were done using self coded software MS-Excel 2010. The Tukey’s post hoc multiple comparison test was done at 0.05 level of significance for the comparisons against control values. Multiple linear regression was performed to determine the relationship between dependent and independent variables and was calculated using following equation:

$$y=a+{b}_{1}{x}_{1}+{b}_{2}{x}_{2}$$

where y = dependent variable, a = y-intercept, b_1_ = partial regression coefficient for x_1_ on y eliminating effect of x_2_, b_2_ = partial regression coefficient for x_2_ on y eliminating effect of x_1,_

x_1_ = independent variable (Fluoride), x_2_ = independent variable (Phthalate).

In order to explain the relative effects of independent variables (fluoride and phthalate) on dependent variable, unitless β-regression coefficients (β1, β2 and β3) were used.

### Ethical approval

This article does not include any studies related with human subjects or animal models performed by any of the authors.

## Supplementary information

Supplementary figure and tables
