# Supplementary material for: Accumulation, morpho-physiological and oxidative stress induction by single and binary treatments of fluoride and low molecular weight phthalates in Spirodela polyrhiza L. Schleiden
Source: Sci Rep. 2019 Dec 27;9:20006. doi: 10.1038/s41598-019-56110-w (PMC6934496; doi:10.1038/s41598-019-56110-w)
Supplement: Supplementary file 1 — Supplementary figure and tables [file 41598_2019_56110_MOESM1_ESM.pdf]

Accumulation, morpho-physiological and oxidative stress induction by single and binary treatments of fluoride and low molecular weight phthalates in *Spirodela polyrhiza* L. Schleiden

[a]Ritika Sharma, [a]Arpna Kumari, [a]Sneh Rajput, [b]Nishu, [a]Saroj Arora, [b]Rajkumar Rampal and Rajinder Kaur[*a]

[*a]*Department of Botanical and Environmental Sciences*

Guru Nanak Dev University, Amritsar-143005

(Punjab), India

[b] *Department of Environmental Science, University of Jammu, 180016, India*

*Corresponding author email id- swab2002@yahoo.com

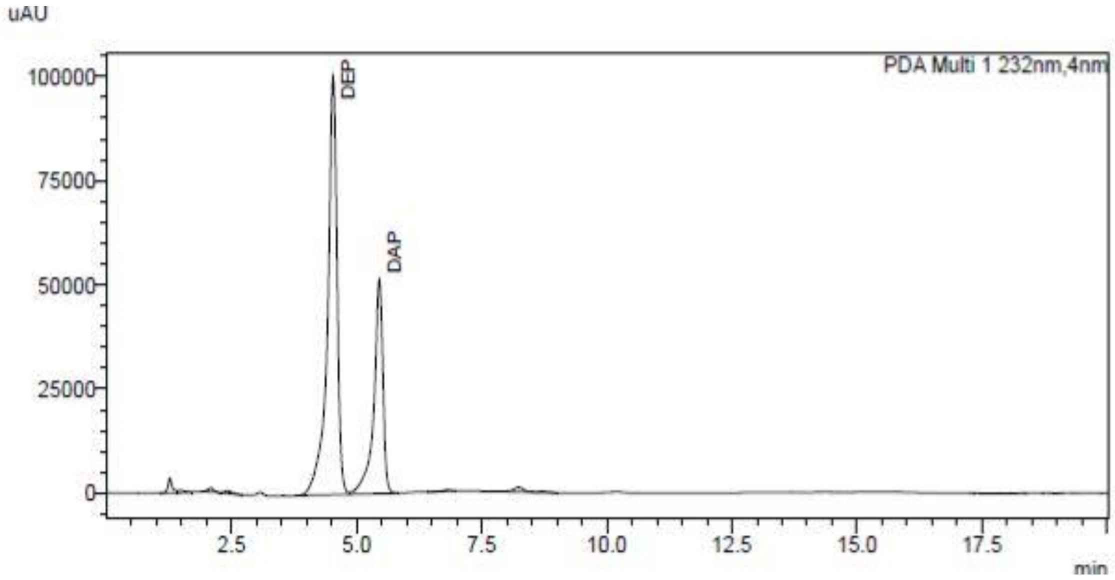

Supplementary Fig 1 a) **Standard chromatogram of DEP and DAP**

Supplementary Table 1 a) Chromatographic conditions of HPLC for method development

**Phthalates**	Diethyl phthalate (DEP); Diallyl phthalate (DAP)
**Column**	C-18 C_{18} having dimensions of 150 × 4.6 mm with pore size of 5µm.
**Column temperature**	38 °C
**Mobile phase**	Acetonitrile (ACN) (Solvent A) and HPLC grade water (Solvent B).
**Gradient elution**	50% solvent A and 50 % solvent B followed by 40 % solvent B for 2.00 min., 30 % solvent B held for 4.00 min, then 35 % solvent B for 5.00 min, followed by 35 % solvent B for 8.00 min and ended at 20.01 min.
**Total run time**	20.01 min
**Flow rate**	0.85 ml/min
**Injection volume**	10 µL
**Detection wavelength**	232 nm

Supplementary Table 1 b) Method development and validation

**Phthalate**	**Linearity range (mg/L)**	**%Recovery**	**%RSD**	**LOD (µL/L)**	**LOQ (µL/L)**	**Regression equation**	**Correlation coefficient (r)**
**Diethyl phthalate (DEP)**	5-400	99.15±2.68	0.28	1.90	5.77	$y = 12022x - 75063$	0.999
**Diallyl phthalate (DAP)**	5-400	98.15±0.99	0.18	0.75	2.30	$y = 12151x - 57090$	0.999
